# Mesenchymal Stem Cells As Guideposts for Nanoparticle-Mediated Targeted Drug Delivery in Ovarian Cancer

**DOI:** 10.3390/cancers12040965

**Published:** 2020-04-14

**Authors:** Buddhadev Layek, Mihir Shetty, Susheel Kumar Nethi, Drishti Sehgal, Timothy K. Starr, Swayam Prabha

**Affiliations:** 1 Department of Experimental and Clinical Pharmacology, University of Minnesota, Minneapolis, MN 55455, USA; buddhadev.layek@ndsu.edu (B.L.); susheel.kumar.nethi@temple.edu (S.K.N.); 2 Department of Obstetrics, Gynecology and Women’s Health (OBGYN), University of Minnesota, Minneapolis, MN 55455, USA; shett036@umn.edu (M.S.); star0044@umn.edu (T.K.S.); 3 Fels Institute for Cancer Research & Molecular Biology, Lewis Katz School of Medicine, Temple University, Philadelphia, PA 19140, USA; 4 School of Pharmacy, Temple University, Philadelphia, PA 19140, USA; tun66699@temple.edu; 5 Masonic Cancer Center, University of Minnesota, Minneapolis, MN 55455, USA

**Keywords:** cancer therapy, mesenchymal stem cells, glycoengineering, ovarian cancer, patient-derived xenograft tumor model, two-step tumor targeting

## Abstract

Nanocarriers have been extensively utilized for the systemic targeting of various solid tumors and their metastases. However, current drug delivery systems, in general, suffer from a lack of selectivity for tumor cells. Here, we develop a novel two-step targeting strategy that relies on the selective accumulation of targetable synthetic receptors (i.e., azide moieties) in tumor tissues, followed by delivery of drug-loaded nanoparticles having a high binding affinity for these receptors. Mesenchymal stem cells (MSCs) were used as vehicles for the tumor-specific accumulation of azide moieties, while dibenzyl cyclooctyne (DBCO) was used as the targeting ligand. Biodistribution and antitumor efficacy studies were performed in both orthotopic metastatic and patient-derived xenograft (PDX) tumor models of ovarian cancer. Our studies show that nanoparticles are retained in tumors at a significantly higher concentration in mice that received azide-labeled MSCs (MSC-Az). Furthermore, we observed significantly reduced tumor growth (*p* < 0.05) and improved survival in mice receiving MSC-Az along with paclitaxel-loaded DBCO-functionalized nanoparticles compared to controls. These studies demonstrate the feasibility of a two-step targeting strategy for efficient delivery of concentrated chemotherapy for treating solid tumors.

## 1. Introduction

Ovarian cancer is the third most common gynecologic cancer in women and the fifth leading cause of cancer deaths among women [1]. The American Cancer Society estimates that in 2020, about 21,750 new cases of ovarian cancer will be diagnosed, and about 13,940 women will die of ovarian cancer in the United States [2]. Women diagnosed with advanced disease (stage IV) have a five-year survival rate of 17%, and this rate has not improved significantly over the last decade [3]. Conventional therapy includes surgical debulking, followed by intravenous platinum- and taxane-based chemotherapy [4,5]. However, a critical problem with traditional chemotherapeutic agents is their severe, dose-limiting toxicities due to a lack of selectivity for tumor tissues.

Encapsulation of chemotherapeutics in nanocarriers, such as liposomes [6,7], nanoparticles [8,9], dendrimers [10,11] or micelles [12,13], which exploit the enhanced permeability and retention (EPR) effect, is the most extensively explored strategy for improving tumor bioavailability and reducing exposure to healthy tissues. However, these systems have had limited success, and only a small fraction (less than 1%) of the injected dose accumulates within the tumor, even in highly permeable tumors [14]. This could be attributed to multiple physiological barriers including endothelial barriers, protein adsorption, phagocytic sequestration and rapid hepatic clearance that hinder the extravasation of nanoparticles into the target tumor tissue [15,16,17,18]. Active targeting utilizes the fact that cancer cells overexpress specific proteins on their surface that can be targeted by conjugating appropriate ligands or antibodies to the nanoparticle surface [19,20,21]. This approach allows for improved retention of nanocarriers in the tumor tissue following their passive accumulation via the EPR effect. Nevertheless, many of these tumor-associated proteins are also expressed on normal cells, making it difficult to achieve a high level of tumor-selectivity by targeting natural membrane proteins. Hence, there is a clear need to develop drug delivery systems that are capable of targeting tumor cells independent of the EPR effect.

To address these problems, we developed a novel two-step targeting strategy that relies on the delivery of mesenchymal stem cells (MSCs) labeled with synthetic functional receptors (azides) to tumor tissues, followed by delivery of drug-loaded nanocarriers targeted to the functional receptors [22]. Previous studies have shown the feasibility of utilizing glycoengineering approaches for tumor targeting [23,24]. These studies also utilized the principle that a synthetic azide sugar is accepted by the CMP-sialic acid biosynthesis machinery in mammalian cells, leading to the biosynthesis and cell surface expression of azido sialic acid containing N_3_-linked glycoproteins [25]. The azide sugar was dosed orally to elicit azide expression in tumor cells. To achieve tumor selectivity, however, it is important to ensure that the synthetic targets are available only in the tumor tissue and not in healthy tissues, which is likely to happen with direct oral delivery of the sugar. Our two-step targeting strategy overcomes this key problem. MSCs were used to introduce artificial azide groups in tumor tissues because of their inherent tumor homing capability [26]. Subsequently, drug-loaded nanoparticles, surface functionalized with dibenzyl cyclooctyne (DBCO) groups, were used to target synthetic azide receptors on the MSCs. We hypothesized that this two-step strategy would enable a significantly higher and more specific delivery of cytotoxic drugs to both, primary tumor and metastatic lesions, resulting in effective inhibition of ovarian tumors. In the present study, the efficacy of two-step targeting was evaluated in an orthotopic, platinum-resistant cell line model (C200) as well as in a patient derived xenograft (PDX) model of ovarian cancer. Our studies show that MSCs engineered to express azide groups on the cell surface localize, and reside, in both the primary tumor, as well as visceral metastases for several weeks. This allows for temporally-controlled, highly selective introduction of synthetic targets in the tumor tissue. Our studies show that these synthetic groups can be targeted with high affinity using small molecule-based targeting ligands. Nanoparticles that target these engineered MSCs accumulate at tumor sites and act as drug depots, slowly releasing the encapsulated drug at the target site, resulting in significantly improved anticancer efficacy. 

## 2. Results

In the current studies, we report the effectiveness of our two-step targeting strategy in improving the therapeutic efficacy of paclitaxel in mouse models of ovarian cancer. We selected paclitaxel (PTX) as a model anticancer drug, because taxanes are one of the front-line adjuvant chemotherapies used for treating ovarian cancer [27,28]. DBCO-PEG functionalized, paclitaxel-loaded nanoparticles (DBCO-PTX NP) were formulated using the FDA-approved, biodegradable and biocompatible polymer, poly(DL–lactide–co–glycolide) (PLGA). These functionalized nanoparticles were then used to target azide bearing MSCs localized in tumor tissues.

### 2.1. Physical Characteristics of Nanoparticles

**Figure 1:**
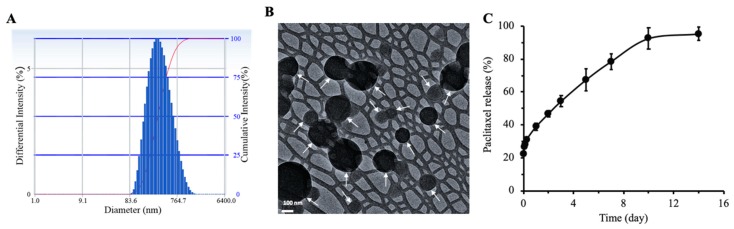
Characterization of DBCO-functionalized, PTX-loaded PLGA nanoparticles (DBCO-PTX NP). (**A**) Particle size distribution as determined by dynamic light scattering. (**B**) A representative TEM image of DBCO-PTX NP. Nanoparticles are shown with white arrows to differentiate from the comb-like mesh background. (**C**) In vitro release profile of PTX from DBCO-PTX NP in MSC complete growth medium supplemented with 10% (*w*/*v*) Captisol^®^ at 37 °C. Data shown is mean ± SD (*n* = 4).

The mean hydrodynamic diameter of DBCO-PTX NP was 331 ± 25 nm with a narrow size distribution as indicated by small PDI values (0.22 ± 0.03) (Figure 1A, Table 1). The zeta potential of DBCO-PTX NP was slightly negative (−11.5 ± 1.3 mV). Transmission electron microscopy (TEM) analysis showed that nanoparticles were discrete and spherical in shape, with a diameter ranging from ~150 nm to 350 nm (Figure 1B). Paclitaxel loading in nanoparticles was 17.2 ± 0.8% (*w*/*w*) with an entrapment efficiency of 73.8 ± 3.6%. 

### 2.2. Kinetics of In Vitro Drug Release

In vitro drug release studies showed an initial burst release over the first 6 h (31% of the encapsulated drug), followed by a steady release up to day 10 (Figure 1C). After 10 days, the drug release reached a plateau and only 3% of the drug was released between days 10 and 14. The total cumulative drug release over 14 days was ~95%.

### 2.3. Biodistribution of Nanoparticles

**Figure 2:**
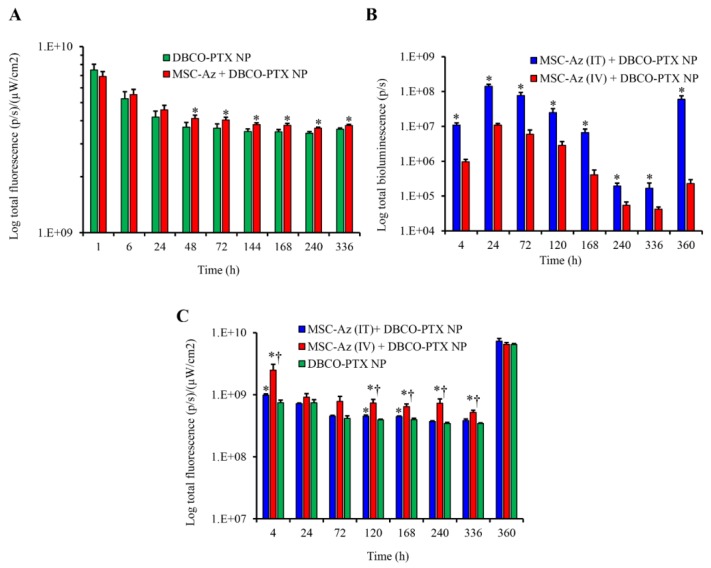
Biodistribution and retention of DBCO-PTX-NIR NP and MSC-Az. (**A**) Biodistribution of DBCO-PTX-NIR NP in C200-Luc orthotopic ovarian tumors (*n* = 6). “*” indicates significantly higher (*p* < 0.05) than DBCO-PTX NP group. (**B**) Biodistribution and or retention of MSC-Luc-Az in PDX ovarian tumor model; *n* = 22 for MSC-Az (IV) and *n* = 18 MSC-Az (IT). At all-time points MSC-IT bioluminescence were significantly higher (*p* < 0.05) than MSC-IV group. “*” indicates significantly higher (*p* < 0.05) than DBCO-PTX NP group. (**C**) Biodistribution of DBCO-PTX-NIR NP in PDX ovarian tumor model; *n* = 22 for MSC-Az (IV) and *n* = 18 for DBCO-PTX NP and MSC-Az (IT) groups. “*” indicates significantly higher (*p* < 0.05) than DBCO-PTX NP group and “†” indicates significantly higher (*p* < 0.05) than MSC-Az + DBCO-PTX NP group.

Tumor accumulation and retention of DBCO-PTX-NIR NP was determined in both the C200 orthotopic ovarian tumors and PDX ovarian tumors based on the fluorescence levels of the encapsulated NIR dye (Figure 2). Representative images are shown in Supplementary Figures S1 and S2.

In the orthotopic C200 model, there was a significant increase in nanoparticle retention in tumors 2 days after injection when MSC-Az cells were co-injected, which persisted for up to two weeks (Figure 2A). This is consistent with the hypothesis that nanoparticles recognized and bound to the azide groups expressed on the MSCs and were retained in the peritoneal cavity for a longer period of time compared to nanoparticles injected without MSCs. In PDX cohort 1, tumor accumulation and retention of MSC-Luc-Az and DBCO-PTX-NIR NPs were determined by in vivo bioluminescence for MSCs (Figure 2B) and fluorescence for nanoparticles (Figure 2C) in individual tumors. As expected, mice receiving an intra-tumoral (IT) injection of MSC-Luc-Az had a higher level of MSC-associated bioluminescence compared to mice that received an intravenous (IV) injection and this difference persisted throughout the study period (Figure 2B). Interestingly, mice that received an IV injection of MSC-Luc-Az had better retention of DBCO-PTX-NIR NPs in tumors, compared to those that received IT injections of MSC-Luc-Az, even though there were fewer MSC-Luc-Az cells in the tumors, based on bioluminescence (Figure 2B,C). One explanation for this finding is that nanoparticles have an enhanced ability to access the MSC’s when they are both administered IV compared to the intratumorally injected MSC’s.

### 2.4. Anticancer Efficacy of Two-Step Targeting Approach

**Figure 3:**
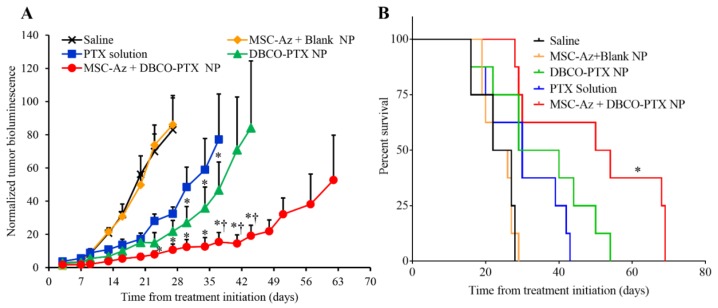
Antitumor efficacy of two-step targeting strategy using glycoengineered MSCs. Mice bearing orthotopic C200-Luc ovarian tumors were intraperitoneally injected with saline; 1 × 10^6^ MSC-Az followed by intraperitoneal injection of DBCO NP (MSC-Az + Blank NP); PTX solution (10 mg/kg, PTX solution); PTX-loaded DBCO NP (equivalent to 10 mg/kg PTX, DBCO-PTX NP); or 1 × 10^6^ MSC-Az followed by intraperitoneal injection of DBCO-PTX NP (equivalent to 10 mg/kg PTX, MSC-Az + DBCO-PTX NP). All animals received respective treatments every 14 d. (**A**) Plot of normalized bioluminescence readings (±SEM; *n* = 8). (*) Indicates significantly different (*p* < 0.05) from PTX solution; † indicates significantly different (*p* < 0.05) from DBCO-PTX NP. (**B**) Kaplan-Meier survival curves for the different treatment groups. Log rank test of MSC-Az + DBCO-PTX NP and control groups yields *p* < 0.0001 (*).

Anti-tumor efficacy of the two-step targeting strategy was evaluated in both C200 and PDX ovarian tumor models. In the C200 orthotopic model, tumors grew equally fast in saline treated and MSC-Az + DBCO NP control groups, indicating that the MSCs by themselves do not affect tumor growth (Figure 3A). Tumor growth was partially inhibited by PTX alone and a slight improvement was seen in mice treated with the same amount of PTX loaded in nanoparticles (DBCO-PTX NPs). The combination of treatment with MSC-Az and DBCO-PTX NPs resulted in the strongest inhibition of tumor growth (*p* < 0.05) compared to all four control groups (Figure 3A). The therapeutic effectiveness of the two-step targeting strategy also manifested in superior survival rates compared to controls (Figure 3B). While the animals in the control groups died between 16–28 days after treatment, the median survival of MSC-Az + DBCO-PTX NP treated animals was 52 days. These results indicate that the two-step strategy (MSC-Az + DBCO-PTX NP) is highly effective in inhibiting the growth of C200-luc tumors, compared to conventional treatments.

**Figure 4:**
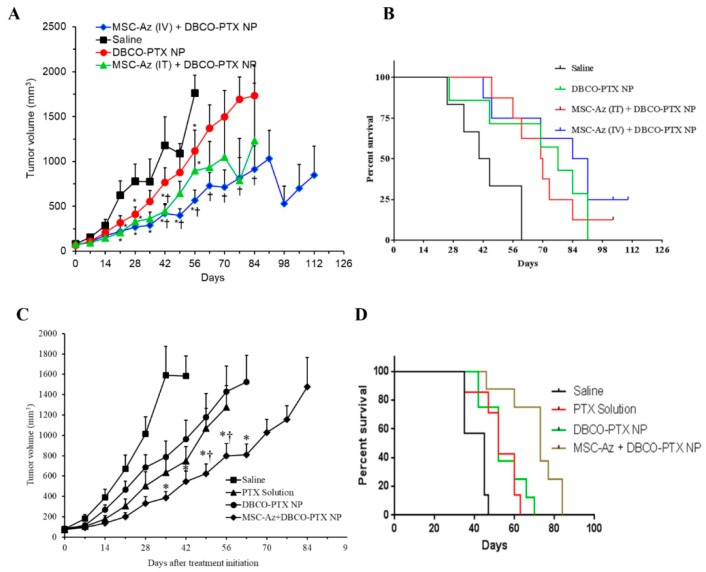
Antitumor efficacy of two-step targeting using glycoengineered MSCs. (**A**,**B**) PDX-bearing mice were intravenously injected with saline; PTX-loaded DBCO functionalized nanoparticles equivalent to 20 mg/kg of PTX (DBCO-PTX NP); 1 × 10^6^ MSC-Az followed by intravenous injection of DBCO-PTX NP (equivalent to 20 mg/kg of PTX) (MSC-Az (IV) + DBCO-PTX NP) and intra-tumoral injection of 0.5 × 10^6^ MSC-Az per tumor followed by intravenous injection of DBCO-PTX NP (equivalent to 20 mg/kg of PTX) (MSC-Az (IT) + DBCO-PTX NP). Mice were dosed with the respective formulation at every 14 days. ‘*’ indicates significantly different (*p* < 0.05) from saline and ‘†’ indicates significantly different (*p* < 0.05) from DBCO-PTX NP group. (**A**) Plot of tumor volume and (**B**) Kaplan–Meier survival curves for the different treatment groups. (**C**,**D**) PDX-bearing mice were intravenously injected with saline; PTX solution (20 mg/kg); PTX-loaded DBCO functionalized nanoparticles equivalent to 20 mg/kg of PTX (DBCO-PTX NP); and 2 × 10^6^ MSC-Az followed by intravenous injection of DBCO-PTX NP (equivalent to 20 mg/kg of PTX) (MSC-Az + DBCO-PTX NP). Mice were dosed with the respective formulation at every 14 days. ‘*’ indicates significantly different (*p* < 0.05) from DBCO-PTX NP group and ‘†’ indicates significantly different (*p* < 0.05) from PTX solution group. (**C**) Plot of tumor volume and (**D**) Kaplan–Meier survival curves for the different treatment groups.

The utility of two-step targeting approach was further evaluated in two PDX models of ovarian cancer (cohort 2 and 3). In PDX cohort 2 (*n* = 29), mice were divided into four groups. Two control groups received either saline or IV injection of DBCO-PTX NPs. Two experimental groups received IV injection of DBCO-PTX NPs along with either an IT or an IV injection of MSC-Az. In this experiment, all three treated groups had significantly reduced tumor growth and improved survival compared to the saline control (Figure 4A; *p* < 0.05). Importantly, the median survival of MSC-Az (IV) + DBCO-PTX NP treated mice was 87 days after treatment initiation, while the median survival of mice in the control and other treatment groups were in the range of 42–77 days (Figure 4B). We also observed tumor regression in 20% (2/8) of the MSC-Az (IV) injected mice, while none of the other groups had tumor regression.

As our previous data indicated that IV injection of MSCs was effective in inhibiting tumors, we repeated the anticancer efficacy of the two-step targeting strategy in a second cohort (cohort 3) comparing the efficacy of IV administration of MSC-Az + DBCO-PTX NP to saline, PTX only, and DBCO-PTX NP controls. We also doubled the dose of MSC-Az in this cohort. We observed significantly reduced tumor growth (Figure 4C; *p* < 0.05) and improved survival (Figure 4D) in the group receiving MSC-Az (IV) + DBCO-PTX NP (median survival 73 days), compared to all other groups (Saline: Median survival 45 days, PTX solution and DBCO-PTX NP: Median survival 52 days).

### 2.5. Immunohistochemical Analysis of Tumors

Pathologic examination of H (Hematoxylin) and E (Eosin) sections identified differences in levels of necrosis within the tumors. Tumors in saline treated animals varied from a relatively low level (score of 1) to high (score of 3), while the PTX-treated animals had considerably higher levels of necrosis (Table 2). The data suggest, and are consistent with, a treatment effect in those animals treated with MSC-Az followed by nanoparticles whether the MSCs were given by the IV or IT routes. The data also suggest that the addition of MSCs increases the therapeutic efficacy of nanoparticles, i.e., 3 of 4 animals with marked necrosis compared to 2 of 4 animals with nanoparticles alone. 

**Figure 5:**
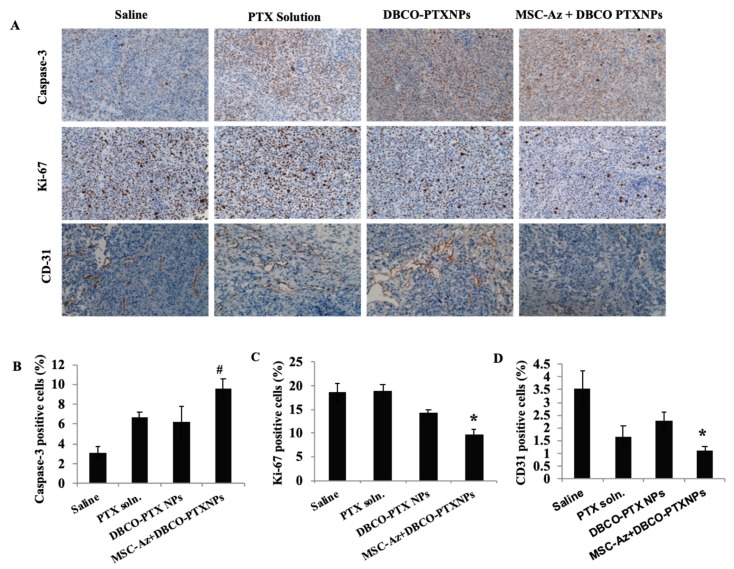
Immuno-histological analysis of tumors. (**A**) Tumor sections were stained for caspase-3 (apoptosis marker), Ki-67 (proliferation marker) and CD-31 (angiogenesis marker). Images were captured at 20× magnification. Quantitative results of (**B**) Caspase-3, (**C**) Ki-67, and (**D**) CD31. Data is represented as mean ± SEM, *n* = 9 images; # *p* < 0.05 compared with saline group and * *p* < 0.05 compared with all treatment groups.

Ki67 expression was high (~80% of cells) in the ovarian tumor cells examined. The high expression of Ki67 likely reflects the high mitotic rate (focally up to 30 mitoses/HMF) in these tumor cells and is consistent with the biology of malignant ovarian tumors. In contrast, caspase-3 was expressed in relatively low numbers of tumor cells. CD31 expression was also not prominent in the tumor tissue; in contrast, it was more prominent in the reactive scirrhous/desmoplastic tissue seen in some animals. However, differences were not observed between the treatment groups with respect to expression of Ki67, Caspase-3, or CD-31 (Table 2). Similar results were observed in the case of second efficacy study (Figure 5).

### 2.6. Real-Time PCR

To determine the presence of MSCs at the tumor site, we performed qRT-PCR to quantitate gene expression of the Thy-1 gene, which is specific to MSCs. The qRT-PCR data confirmed the presence of MSCs in the tumor tissues (Table 3).

## 3. Discussion

The therapeutic efficacy of conventional chemotherapeutics is often limited by poor solubility, unfavorable pharmacokinetic profiles, and lack of tumor selectivity, which eventually leads to severe toxicities [29]. These challenges can be overcome by packaging the drugs in nanocarriers, which enables their improved tumor delivery through the passive tumor accumulation of the nanocarriers. However, the EPR-mediated tumor targeting is highly inefficient, especially in low-permeability tumors and in less accessible tumors (e.g., ovarian, brain, bone, etc.) [30]. Retention of nanocarriers in tumors can be further improved through active targeting, which relies on the biological interaction between the nanocarrier-conjugated targeting ligands and cell-surface receptor proteins or antigens. Numerous ligands have been identified and investigated for facilitating active targeting of nanocarriers for enhanced retention at the target tissue, and improved uptake by the desired cells [31,32,33]. However, nanocarriers that are intended for active targeting have to passively accumulate in tumors first. Furthermore, many of these receptors are also expressed on healthy cells and offset the advantages of active targeting.

The two-step targeting strategy investigated here relies on the introduction of synthetic receptors (i.e., azide functional groups) on MSCs, which naturally migrate to tumors, resulting in a high concentration of the azide groups in the tumor [34]. The second step is the introduction of DBCO-functionalized drug-loaded nanoparticles that have a high affinity for the azide groups. The click reaction between azide receptors and DBCO is relatively fast, highly specific, catalyst-free, and can proceed under physiological conditions [23,35,36]. Additionally, azide and DBCO moieties are relatively small and biologically inert, facilitating the incorporation of multiple azide groups on the cell surface and subsequent click reaction [37]. We utilized MSCs as vehicles to introduce azide moieties in the tumor tissues due to their favorable characteristics. MSCs exist in almost all tissues and can be easily isolated from various human tissues (e.g., bone marrow, adipose tissue, muscle, lung, liver, and umbilical cord) with minimal invasion [38,39,40]. Importantly, MSCs possess several surface markers (e.g., CXCR4, CXCR12, and CCL2) that allow them to home to the tumor sites in response to chemokines secreted by the tumor cells [41,42]. 

Previous studies have shown the feasibility of utilizing glycoengineering approaches for tumor targeting [24,36]. These studies also utilized the principle that azide sugar is accepted by the CMP-sialic acid biosynthesis machinery in mammalian cells, leading to the biosynthesis and cell surface expression of azido sialic acid containing N_3_-linked glycoproteins [43]. In those studies, the azide sugar was administered systemically to the animals to elicit azide expression directly in tumor cells. To achieve tumor selectivity, however, it is crucial to ensure that the synthetic azides are available only in the tumor tissue and not in healthy tissues, which is likely to happen with systemic delivery of the sugar. Our studies show that this limitation can be overcome by the two-step targeting strategy. 

In our previous studies, we optimized the glycoengineering protocol to achieve high azide expression on MSCs without affecting their viability or tumor homing characteristics [22,44]. MSCs were found to not be sensitive to paclitaxel induced cytotoxicity [45]. In addition, these studies also demonstrated improved tumor growth inhibition in a platinum-sensitive orthotopic ovarian tumor model [22,44]. Tumor xenografts established from immortalized cell lines are easy to develop, reproducible, easy to measure by incorporating bioluminescence, and relatively inexpensive [46]. Therefore, they are frequently used in initial proof-of-efficacy trials. However, a major limitation of traditional xenograft models is that the genetics and histology of cell lines is altered during the multiple rounds of in vitro passaging, rendering these models less predictive of therapeutic success [47,48]. PDX models, on the other hand, are established by the direct transplantation of human tumors into immune-compromised mice and maintained by serial passaging into mice, without ever being subjected to in vitro growing conditions. These models, at low passages, maintain the original histologic [49,50] and genomic [51] characteristics of the tumors from which they were derived. Consequently, PDX models serve as better surrogates for patients and are better at predicting the response to treatment [52]. Thus, in the present study, we used both models to evaluate our two-step targeting strategy. We obtained the same results in both models, indicating the two-step approach is effective in highly relevant pre-clinical models.

Ovarian cancer can be divided into two types based on deficient DNA repair mechanisms, with one group being homologous repair deficient (HRD positive) and the other group being homologous repair sufficient (HRD negative). A new therapy using PARP inhibitors was recently approved for treating ovarian cancer, and this therapy is considerably more effective in HRD ovarian cancers [53,54,55]. Interestingly, in our study, the PDX model used in cohort 2 (Figure 4A,B) was HRD positive due to a germline mutation in BRCA1, while the PDX model used in cohort 3 (Figure 4C,D) was likely HRD negative, as there were no detectable mutations in BRCA1 or 2. The treatment response in both of these models was similar, suggesting our method of tumor control is not affected by the tumor intrinsic HRD status.

Tumor-specific delivery of cytotoxic drugs is a key requirement for the success of chemotherapy. Our studies show that DBCO-functionalized nanoparticles are retained in tumors at a significantly higher concentration in the case of mice that received MSC-Az compared to the mice that did not receive MSC-Az (Figure 2A). Moreover, the enhanced retention of nanoparticles persisted for two weeks. The high nanoparticle retention level in the MSC-Az-treated group could be attributed to the lower rate of elimination of nanoparticles that recognized and bound to MSC-Az. The biodistribution profiles of DBCO-functionalized nanoparticles and MSC-Az were also confirmed in PDX tumor-bearing mice. As expected, we observed a persistent higher accumulation and retention of MSC-Luc-Az in the case of intratumorally injected mice (Figure 2B). In contrast, mice receiving intravenous injection of MSC-Luc-Az showed higher retention of nanoparticles in tumors compared to mice that received intratumoral injections of MSC-Luc-Az (Figure 2C). We speculate that MSC-Luc-Az administered intravenously are more accessible than those administered intratumorally to intravenously injected nanoparticles. This would lead to a greater interaction of DBCO-functionalized nanoparticles with MSC-Luc-Az via click chemistry, resulting in higher nanoparticle retention in the tumor. Overall, the results of biodistribution studies clearly demonstrated that the two-step targeting approach leads to relatively higher and longer retention of DBCO-functionalized nanoparticles in the tumor tissue. 

The effectiveness of two-step targeting strategy in improving the therapeutic efficacy of paclitaxel was established in the orthotopic metastatic cell-line ovarian tumor model as well as two PDX models of ovarian cancer. The enhanced antitumor efficacy of the two-step targeting strategy could be attributed to the higher and longer retention of DBCO-functionalized nanoparticles in the tumor tissue. Tumor-intrinsic mechanisms of chemotherapy resistance are likely to be important in any therapy employing cytotoxic chemotherapies. Interestingly, in a previous study, we found that our two-step approach was highly effective at inhibiting growth of a platinum-sensitive ovarian cancer cell line (MA148) derived from a patient who responded to platinum-based chemotherapy [22]. In contrast, in our current study, we used an ovarian cancer cell line that was generated by continuous exposure to chemotherapy, and represents a platinum-resistant cell line [56,57]. This difference in intrinsic chemotherapy resistance was reflected in a less-robust level of growth inhibition using the two-step approach in the highly resistant C200 cell line (compare Figure 3A this publication to Figure 7A in [22]).

Paclitaxel’s primary mechanism of cytotoxicity is attributed to its ability to bind tubulin and stabilize microtubules, resulting in mitotic arrest and subsequent cell death [58,59]. However, there is evidence that paclitaxel may employ alternative methods of cell-killing, independent of mitotic arrest [60]. The mechanisms of internalization, release and functional activity of paclitaxel-loaded nanoparticles in tumor cells are not well-understood, especially within a physiologically intact tumor microenvironment. To begin to understand these mechanisms, we stained tumors for markers of proliferation (Ki-67), apoptosis (cleaved-caspase-3), angiogenesis (CD31) and evaluated levels of necrosis (Table 2). When we compared paclitaxel delivery using nanoparticles (both with or without the addition of MSCs) to saline controls we found no changes in apoptosis or angiogenesis markers in the tumors, while there was a slight decrease in the proliferation marker Ki-67 and an increase in necrosis (Table 2). This is somewhat surprising, as paclitaxel is known to trigger apoptosis in ovarian cancer due to its capacity to decrease mitochondrial membrane potential [59]. We did not detect any noticeable differences in the markers or necrosis score, when comparing free nanoparticles to nanoparticles, combined with MSCs, suggesting our two-step delivery method does not affect paclitaxel’s mechanism of cell cytotoxicity when delivered using nanoparticles.

## 4. Materials and Methods

### 4.1. Materials

Paclitaxel was purchased from TCI America (Portland, OR, USA). Ester-terminated poly(DL–lactide–*co*–glycolide) (50:50) (PLGA), with an inherent viscosity of 0.55–0.75 dL/g, was purchased from Lactel Absorbable Polymers (Birmingham, AL, USA). Poly (D, L lactide)-b-poly(ethylene glycol)-amine (PLA-PEG-NH_2_, PLA molecular weight: 10,000 and PEG molecular weight: 5000 Da) was obtained from Nanosoft Polymers (Winston-Salem, NC, USA). Dibenzyl cyclooctyne (DBCO)-sulfo-NHS ester, *N*-azidoacetylmannosamine-tetraacylated (Ac_4_ManNAz), and DBCO-sulforhodamine B were obtained from Click Chemistry Tools (Scottsdale, AZ, USA). Near infrared (NIR) fluorescent dye SDB5491 was procured from H.W. Sands Corp. (Jupiter, FL, USA). Ammonium acetate, chloroform, and polyvinyl alcohol (PVA) were purchased from Sigma (St. Louis, MO, USA). HPLC grade acetonitrile and methanol were purchased from Fisher Scientific (Pittsburgh, PA, USA). D-Luciferin potassium salt was procured from Gold Biotechnology (Saint Louis, MO, USA). Fetal bovine serum (FBS) was obtained from Atlanta Biologicals Inc. (Flowery Branch, GA, USA). Penicillin/streptomycin was purchased from Bioexpress (Kaysville, UT, USA). Dulbecco’s phosphate buffered saline (DPBS), RPMI 1640, and trypsin-EDTA solution were procured from Life Technologies (Grand Island, NY, USA). Human epithelial ovarian carcinoma cell line C200 was a kind gift from Dr. Sundaram Ramakrishnan (Department of Surgery, University of Miami, Miami, FL, USA). The C200 cell line is a highly platinum resistant isogenic cell line generated from A2780 ovarian cancer cells [56,57]. Bone marrow-derived human MSCs and human mesenchymal stem cell medium (MSCM) were purchased from ScienCell Research Laboratories (Carlsbad, CA, USA).

### 4.2. Preparation of PLA-PEG-DBCO

The synthesis of PLA-PEG-DBCO co-polymer was performed by reacting PLA-PEG-NH_2_ with DBCO-sulfo-N-hydroxysuccinimide ester. PLA-PEG-NH_2_ (60 mg) was dissolved in 0.5 mL tetrahydrofuran and DBCO-sulfo-NHS ester (10 moles per mole of PLA-PEG-NH_2_) was dissolved in 5 mL phosphate buffered saline. The PLA-PEG-NH2 solution was added dropwise to the DBCO-sulfo-NHS ester solution and the reaction was continued overnight while stirring at room temperature. The resulting co-polymer was purified by dialysis against deionized water for two days using dialysis cassettes (3.5 kDa MWCO), followed by freeze-drying. Formation of PLA-PEG-DBCO co-polymer was verified by ^1^H NMR spectroscopy.

### 4.3. Preparation of Nanoparticles

Paclitaxel and NIR dye SDB5491 loaded DBCO-functionalized PLGA nanoparticles (DBCO-PTX NIR NP) were prepared by interfacial activity assisted surface functionalization technique [22,44,61]. In brief, PLGA (32 mg), paclitaxel (8 mg), and SDB5491 (0.25 mg) were dissolved in chloroform (1 mL) and added to aqueous PVA solution (7.5 mL, 2.5% *w*/*v*). The mixture was probe sonicated at 18–21 W for 5 min over an ice bath (Sonicator XL, Misonix, NY, USA) to form an oil-in-water emulsion. The block co-polymer PLA-PEG-DBCO (8 mg) was dissolved in chloroform (0.2 mL) and added dropwise to the above emulsion with continuous stirring. The emulsion was further stirred overnight (~18 h) under ambient conditions, followed by 1 h stirring under vacuum for complete removal of chloroform. Nanoparticles were collected by ultracentrifugation (35,000 rpm for 35 min at 4 °C, Optima XPN-80 Ultracentrifuge, Rotor type: 50.2 Ti, Beckman Coulter, Brea, CA, USA) and resuspended in deionized water. This washing procedure was repeated two additional times to remove unencapsulated drug, free polymer, and residual PVA. Nanoparticle suspension was then centrifuged (1000 rpm for 5 min, Allegra X-30R Centrifuge, Rotor type: SX 4400, Beckman Coulter Inc., Brea, CA, USA) to remove large aggregates, and the supernatant was lyophilized (Labconco, FreeZone 4.5, Kansas City, MO, USA). Drug-free blank nanoparticles were fabricated similarly. 

### 4.4. Characterization of Nanoparticles

Particle size, polydispersity index (PDI), and zeta potential of nanoparticles were determined by Delsa Nano C particle analyzer (Beckman Coulter, CA, USA). The samples were dispersed in deionized water and analyzed in replicates of four at 25 °C. The morphology of nanoparticles was observed using TEM. The colloidal dispersion in deionized water (~3 µL, 0.1 mg/mL) was dropped onto a copper grid covered with a thin carbon layer (400 mesh, Ted Pella Inc., Redding, CA, USA) and dried overnight under ambient conditions. Images were captured using a FEI Tecnai G2 F30 S-TWIN TEM operating at 300 kV (Gatan, Pleasanton, CA, USA).

To determine entrapment efficiency and drug loading, paclitaxel loaded nanoparticles were dispersed in methanol. Drug was extracted overnight at room temperature with gentle agitation using a rotary extractor. Nanoparticle debris was separated from the extract by centrifugation at 13,000 rpm for 15 min (Allegra X-30R Centrifuge, Rotor type: FX 301.5). The amount of paclitaxel in the supernatant was determined by HPLC as described previously [22].

The entrapment efficiency (EE), and drug loading (DL), were calculated using the following equations, respectively:

$$Entrapment\;efficiency\;\left(\%\right)=\frac{\mathrm{Amount}\;\mathrm{of}\;\mathrm{drug}\;\mathrm{in}\;\mathrm{nanoparticles}}{\mathrm{Total}\;\mathrm{amount}\;\mathrm{of}\;\mathrm{drug}\;\mathrm{added}}\times 100$$

$$Drug\;loading\;\left(\%\right)=\frac{\mathrm{Amount}\;\mathrm{of}\;\mathrm{drug}\;\mathrm{in}\;\mathrm{nanoparticles}}{\mathrm{Amount}\;\mathrm{of}\;\mathrm{drug}\;\mathrm{loaded}\;\mathrm{nanoparticles}}\times 100$$

### 4.5. In Vitro Drug Release

In vitro release kinetics of paclitaxel from nanoparticles was performed in complete cell culture medium supplemented with 10% *w/v* Captisol^®^ (Cydex Pharmaceuticals, Lawrence, KS, USA) [62]. Nanoparticle dispersions were incubated in a shaker at 37 °C and 100 rpm. At predetermined time points (1 h, 2 h, 4 h, 6 h, 1 d, 2 d, 3 d, 5 d, 7 d, 10 d, and 14 d), the released drug was separated from nanoparticles by centrifuging at 13,000 rpm for 15 min. The supernatant was analyzed for paclitaxel content by HPLC.

### 4.6. Cell Culture

C200 cells were grown in RPMI 1640 medium supplemented with 10% FBS, 0.5 µg/mL human insulin, 100 µg/mL streptomycin, and 100 U/mL penicillin. MSCs were grown in MSCM medium supplemented with 5% FBS, 100 µg/mL streptomycin, 100 U/mL penicillin, and 1× MSC growth supplement (ScienCell Research Laboratories, Carlsbad, CA, USA). All the cells were maintained at 37 °C and 5% carbon dioxide in a humidified incubator.

### 4.7. Generation of Luciferase-Expressing C200 (C200-Luc) Cells and MSCs (MSC-Luc)

Luciferase transduction of MSCs and C200 cells were carried out using Firefly Luciferase Lentifect™ Purified Lentiviral Particles (GeneCopoeia, Rockville, MD, USA). Briefly, 0.5 × 10^6^ cells were seeded in a 25 cm^2^ culture flask in 4 mL RPMI complete medium and incubated at 37 °C with 5% CO_2_ overnight. Next day, the cells were treated with viral suspension diluted in a medium containing 50 µg/mL protamine sulfate at the multiplicity of infection of 10. Cells were incubated for 24 h at 37 °C and 5% CO_2_. Post-transduction, the cells were cultured in complete growth medium supplemented with 3 µg/mL puromycin to select stably transduced cells.

The luciferase activity of C200-luc and MSC-luc cells was measured using IVIS Spectrum In Vivo Imaging System (Caliper Life Sciences) upon D-luciferin exposure. Briefly, cells were plated in 6 well plates at different cell densities, ranging from 6.25 × 10^3^ to 1 × 10^5^ cells/well. After 4 h of incubation, cells were treated with D-luciferin diluted in complete growth media (150 µg/mL final concentration). Bioluminescent images were recorded after a 10 min exposure to D-luciferin and analyzed using Living Image software version 4.2 (Caliper Life Sciences). 

### 4.8. Generation of Azide Labeled MSCs (MSC-Az)

MSCs expressing azide functional groups (MSC-Az) were prepared by culturing MSCs in presence of Ac_4_ManNAz sugar containing growth medium (20 µM) for 3 days [22]. The presence of azides on the cell surface was confirmed by incubating MSC-Az with DBCO-sulforhodamine B (20 µM final, 1 h). Cells were washed 3 times with DPBS and visualized using a fluorescence microscope.

### 4.9. Animal Tumor Models

All animal experiments were approved by the Institutional Animal Care and Use Committee at the University of Minnesota (Protocol ID: 1702-34600A and 1605-33821A). Collection of human samples for generation of PDX models was approved by the Institutional Review Board of the University of Minnesota (Protocol ID: 1408M52905). Female athymic nude (Crl:NU(NCr)-Foxn1nu) mice, five weeks old, were purchased from Charles River Laboratories (Wilmington, MA, USA).

#### 4.9.1. Generation of Orthotopic Ovarian Tumor Model

Orthotopic ovarian tumors were developed in athymic nude mice by intraperitoneal injection of 1 × 10^6^ C200-luc cells dispersed in 200 µL DPBS. Tumor progression was monitored through in vivo bioluminescence imaging. At regular intervals, mice were injected intraperitoneally with D-luciferin potassium salt (150 mg/kg) and bioluminescence images were captured using IVIS Spectrum In Vivo Imaging System.

#### 4.9.2. Generation of PDX Tumor Models

PDX ovarian tumors were generated by subcutaneous transplantation of human ovarian tumor tissues into female athymic nude mice. Tumor tissue samples collected from the operating room were processed to remove necrotic tissue and cut into 2 mm^3^ cubes using sterile forceps and scalpel. Each mouse typically received two transplants in flanks by making an incision, opening a small pouch, inserting the tumor tissue, and closing the pouch using a wound clip. The clips were removed once the wounds healed. To generate the cohorts of mice used in this study, a large tumor from a PDX mouse was harvested and cut into several pieces for serial transplantation into the required number of mice. Tumor dimensions were measured with a digital slide caliper and tumor volumes were calculated according to the following equation: volume = (L × L × W)/2, where L is tumor length and W is tumor width.

#### 4.9.3. Biodistribution and Efficacy Studies were Performed in the PDX Ovarian Tumor Models as Described Below

PDX Cohort 1: The first PDX model was established from an omental metastasis harvested from a patient with a germline BRCA1 mutation during her primary debulking. Pathological staging determined that the tumor was a stage IIIC high grade serous ovarian cancer. The tumor was considered platinum sensitive as no evidence existed of recurrence within 6 months of the final chemotherapy treatment. A cohort of 36 PDX mice were generated using a serial passaged tumor from a PDX mouse established with this patient’s tumor.

PDX Cohort 2: This cohort was established using a second PDX mouse from the same patient described in cohort 1. A total of 29 PDX mice were generated in this cohort. 

PDX Cohort 3: The third PDX model was established from an omental metastasis harvested from a second patient who did not have a detected germline BRCA1 or BRCA2 mutation during her primary debulking. Pathological staging determined that the tumor was a stage IV high grade serous ovarian cancer. The tumor was considered platinum resistant due to recurrence of disease within 6 months of the final chemotherapy treatment. A cohort of 30 mice was generated using a serial passaged tumor from a PDX mouse established with this patient’s tumor. 

### 4.10. Biodistribution of Nanoparticles

#### 4.10.1. Orthotopic Ovarian Tumor Model

A cohort of tumor-bearing mice (*n* = 12) were randomly divided into two groups receiving either paclitaxel and NIR dye loaded DBCO-functionalized nanoparticles (equivalent to 10 mg/kg paclitaxel, “DBCO-PTX-NIR NP”) or 1 × 10^6^ MSC-Az followed by intraperitoneal injection of equivalent amount DBCO-PTX-NIR NP (MSC-Az + DBCO-PTX-NIR NP). Mice were re-dosed with the respective treatments on day 14. The biodistribution of dye loaded nanoparticles was monitored using an IVIS Spectrum In Vivo Imaging System (λ_ex_: 745 and λ_em_: 820 nm) at different time points after treatment initiation. The fluorescence images were captured and processed using Living Image® software (PerkinElmer). The total fluorescence intensity from the region of interest (ROI) was determined and plotted on a logarithmic scale.

#### 4.10.2. PDX Tumor Models

Mice from Cohort 1 were divided into three groups (*n* = 12/group). When tumor volume reached ~150 mm^3^, animals in group 1 received intravenous (IV) tail vein injections of 2 × 10^6^ MSC-Luc-Az, animals in group 2 received intra-tumoral (IT) injections of 1 × 10^6^ MSC-Luc-Az per tumor, and animals in group 3 did not receive MSCs. One hour following MSC-Luc-Az injection, DBCO-PTX-NIR NPs (equivalent to 20 mg/kg of paclitaxel) were injected intravenously into all 36 animals. All animals were re-dosed with the same formulation on day 14. Animals were imaged for both bioluminescence and fluorescence (λ_ex_: 745 and λ_em_: 820 nm) using an IVIS Spectrum In Vivo Imaging System at different time points after initial nanoparticle administration (4 h, 24 h, 3 d, 5 d, 7 d, 10 d, 14 d, and 15 d). For bioluminescence imaging, each mouse was injected with 200 µL of 15 mg/mL D-luciferin potassium salt intraperitoneally, 15 min before imaging. Each tumor was imaged separately, and the tumor accumulation of MSC-Luc-Az and nanoparticles was calculated based on bioluminescence and fluorescence levels, respectively. Both the bioluminescence and fluorescence images were captured and analyzed using Living Image® software (PerkinElmer). The total bioluminescence and fluorescence intensities from the regions of interest (ROIs) were determined and plotted on a logarithmic scale.

### 4.11. Anticancer Efficacy of Two-Step Targeting Approach

#### 4.11.1. Efficacy against C200-Luc Tumors

Mice bearing orthotopic ovarian C200-luc tumors were randomly divided into five groups and were injected intraperitoneally with either DPBS (Saline); paclitaxel solution (10 mg/kg, “PTX solution”); paclitaxel-loaded DBCO-PTX NP (equivalent to 10 mg/kg of paclitaxel, “DBCO-PTX NP”); 1 × 10^6^ MSC-Az followed by intraperitoneal injection of DBCO-PTX NP (equivalent to 10 mg/kg paclitaxel, “MSC-Az + DBCO-PTX NP”); or 1 × 10^6^ MSC-Az followed by intraperitoneal injection of DBCO-functionalized drug free nanoparticles (same amount of nanoparticles as in the case of MSC-Az + DBCO-PTX NP, “MSC-Az + Blank NP”). All animals were re-dosed with the respective treatments every 14 days. Tumor growth was monitored by measuring bioluminescence at different time intervals and represented as normalized bioluminescence. The day when the treatments were initiated was considered day 0. Animals were euthanized when they showed signs of distress or moribundity.

#### 4.11.2. Efficacy against PDX Tumors

Mice with PDX tumors (Cohort 2, *n* = 29) were randomly divided into four groups receiving intravenous injection of saline (*n* = 6), DBCO-PTX NP (equivalent to 20 mg/kg of paclitaxel, *n* = 7), intravenous injection of 1 × 10^6^ MSC-Az followed by intravenous injection of DBCO-PTX NP (equivalent to 20 mg/kg of paclitaxel, MSC-Az-IV + DBCO-PTX NP; *n* = 8), and intratumoral injection of MSC-Az (0.5 × 10^6^ per tumor) followed by intravenous injection of DBCO-PTX NP (equivalent to 20 mg/kg of paclitaxel, MSC-Az-IT + DBCO-PTX NP; *n* = 8). All animals were re-dosed with the respective treatments every 14 d. Tumor dimensions were measured with a digital slide caliper on regular intervals. The day when treatments were initiated was considered day 0. Animals were euthanized when they showed signs of distress, moribundity or tumor diameter exceeded 2 cm.

#### 4.11.3. Effect of Increased Dose of MSC against PDX Tumors

We determined the effect of increased dose of MSC-Az (1 × 10^6^ MSC-Az vs. 2 × 10^6^ MSC-Az) in inhibiting tumors. Mice with PDX tumors (Cohort 3) were randomly divided into four groups receiving intravenous injection of saline (*n* = 7), paclitaxel solution (PTX solution; *n* = 7), DBCO-PTX NP (equivalent to 20 mg/kg of paclitaxel; *n* = 8), and intravenous injection of 2 × 10^6^ MSC-Az followed by intravenous injection of DBCO-PTX NP (equivalent to 20 mg/kg of paclitaxel, MSC-Az + DBCO-PTX NP; *n* = 8). All animals were re-dosed with the respective treatments every 14 days. Tumor growth was monitored by measuring tumor dimensions using a digital slide caliper as described above.

### 4.12. Immunohistochemical Analysis of Tumors

To monitor the effect of treatment on angiogenesis, inhibition of cell proliferation, and induction of apoptosis, we performed immunohistochemical analysis (IHC) of the tumor tissues. At the end of the study, tumor tissues were collected and fixed in 4% (*v*/*v*) formaldehyde solution for 24 h followed by storage in 70% ethanol. The fixed tumors were processed, paraffin-embedded, and slides were prepared with 4-mm-thick slices. Slides were stained for Ki-67 (for tumor proliferation), cleaved caspase-3 (for apoptosis), and CD31 (for angiogenesis). Slides were imaged using EVOS M5000 Imaging System (ThermoFisher Scientific, Waltham, MA, USA) and images (*n* = 9, for each group) were quantified for percentage positive tumor cells for caspase-3, Ki-67 and CD31 using ImageJ software. Tumor samples were also examined to assess the degree of necrosis. 

### 4.13. Real Time PCR

To determine the presence of MSCs at the tumor site, we performed real-time polymerase chain reaction (RT-PCR) of the representative tumor tissues collected from the PDX efficacy study (cohort 2). Total RNA was isolated from tumor tissues using the RNeasy Plus Universal Kit (Qiagen, Valencia, CA, USA) according to the manufacturer’s protocols. The final extracted RNA sample was used to quantify Thy-1 gene expression by quantitative RT-PCR (QuantStudio 7 Flex Real-Time PCR System, Thermo Fisher Scientific) using the following primers: TCATGTCCCTGTGACTGGTG (forward) and GGATCTCTGCACTGGAACTTG (reverse) with Probe 25 from the Roche UPL system. The mRNA levels were normalized against expression of glyceraldehyde-3-phosphate dehydrogenase (GAPDH) as a housekeeping gene.

### 4.14. Statistical Analysis

Statistical analyses were performed using Student’s t-test and one-way ANOVA, followed by Bonferroni-Holm method for comparison between individual treatment groups. Log-rank test was performed, in order to compare survival among different treatment groups. A *p*-value of less than 0.05 was considered significant.

## 5. Conclusions

Traditional active-targeting strategies rely on the biological interaction between the nanocarrier-conjugated affinity ligands and cell-surface receptor proteins. However, most of these receptors are also expressed on healthy tissues, and thus, offset the tumor-selective drug accumulation. Our studies show that the two-step targeting strategy overcomes this critical issue by creating high-affinity synthetic receptors in the tumors. Most importantly, the two-step targeting approach leads to relatively greater and longer retention of DBCO-functionalized nanoparticles in the tumor tissue, which leads to enhanced antitumor efficacy.

## Figures and Tables

**Table 1:** Mean particle size, PDI, zeta potential, drug loading, and entrapment efficiency of DBCO-PTX NP. Data shown is mean ± SD (*n* = 6).

Parameters	Values
Particle size (nm)	331 ± 25.1
PDI	0.22 ± 0.03
Zeta potential (mV)	−11.5 ± 1.3
Drug loading (%)	17.2 ± 0.8
Entrapment efficiency (%)	73.8 ± 3.6

**Table 2:** Histological analysis of tumors collected at the end of the efficacy study. Tumor tissues were evaluated semi-quantitatively and graded from 1–4 (1 ≤ 25% of the tumor cells positive, 2 ≥ 25 to 50% positive, 3 ≥ 50 to 75% and 4 ≥ 75 to100% cell positivity).

Group/Score	Ki-67				Caspase-3				CD31				Necrosis Score			
	1	2	3	4	1	2	3	4	1	2	3	4	1	2	3	4
Saline	0	0	0	3	3	0	0	0	3	0	0	0	0	2	1	0
DBCO-PTX NP	0	1	1	2	4	0	0	0	4	0	0	0	0	1	1	2
MSC-Az (IV) + DBCO-PTX NP	0	0	2	2	4	0	0	0	4	0	0	0	0	0	1	3
MSC-Az (IT) + DBCO-PTX NP	0	1	1	2	4	0	0	0	4	0	0	0	0	1	0	3

**Table 3:** Analysis of relative gene expression data using Real-Time Quantitative PCR. Data represents mean ± SD (*n* = 4). Untreated tumors were used as blank control.

Sample	Expression Fold Change (2^−∆∆Ct^)
MSC-Az (IV) + DBCO-PTX NP	1.90 ± 0.92
MSC-Az (IT) + DBCO-PTX NP	1.81 ± 0.56

## Supplementary Materials

The following are available online at https://www.mdpi.com/2072-6694/12/4/965/s1, Figure S1: Biodistribution and retention of DBCO-PTX-NIR NP in C200-Luc orthotopic ovarian tumor-bearing mice following intraperitoneal injection of nanoparticles with or without MSC-Az. Representative fluorescence images at different time intervals are shown with the corresponding fluorescence intensity scale [4.0 × 10^7^–2.0 × 10^8^ (photons/s)/(µW/cm^2^)], Figure S2: Biodistribution and retention of DBCO-PTX-NIR NP in PDX ovarian tumor model. Representative fluorescence images at different time intervals are shown with the corresponding fluorescence intensity scale [5 × 10^7^–2.0 × 10^8^ (photons/s)/(µW/cm^2^)].
