# Supplementary material for: Mesenchymal Stem Cells As Guideposts for Nanoparticle-Mediated Targeted Drug Delivery in Ovarian Cancer
Source: Cancers (Basel). 2020 Apr 14;12(4):965. doi: 10.3390/cancers12040965 (PMC7226169; doi:10.3390/cancers12040965)
Supplement: Supplementary file 1 [file cancers-12-00965-s001.pdf]

SUPPLEMENTARY FIGURE

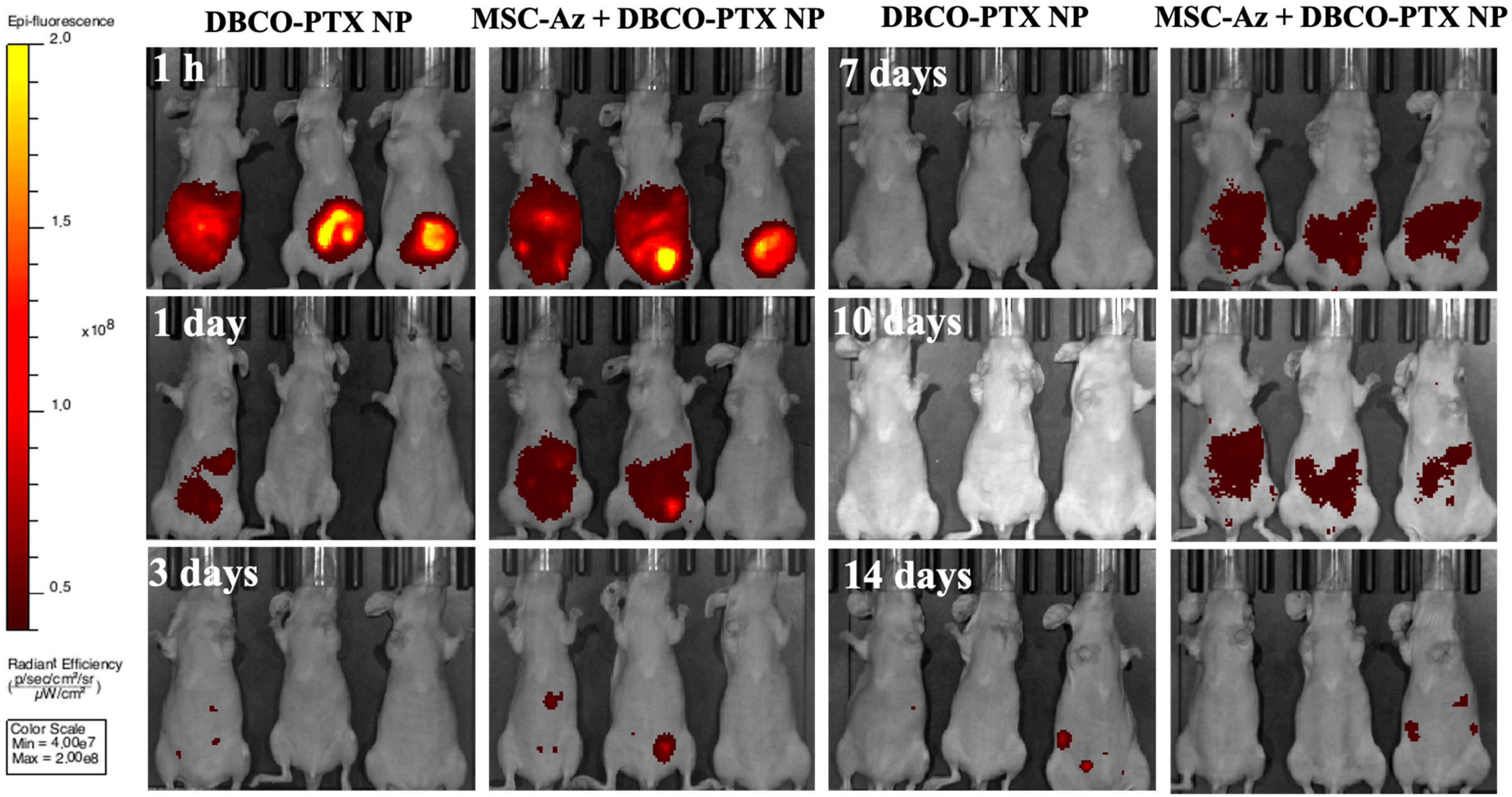

Figure S1. Biodistribution and retention of DBCO-PTX-NIR NP in C200-Luc orthotopic ovarian tumor-bearing mice following intraperitoneal injection of nanoparticles with or without MSC-Az. Representative fluorescence images at different time intervals are shown with the corresponding fluorescence intensity scale [4.0×10^7–2.0×10^8 (photons/s)/(μW/cm^2)].

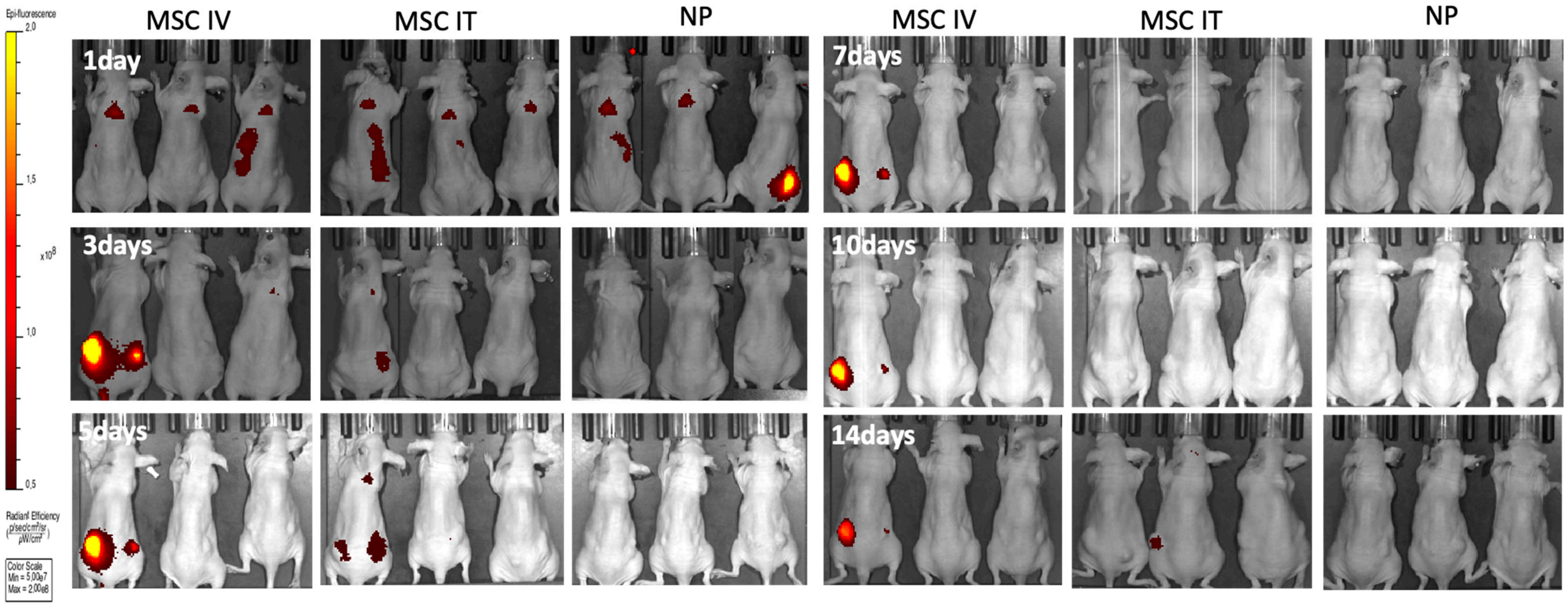

Figure S2. Biodistribution and retention of DBCO-PTX-NIR NP in PDX ovarian tumor model. Representative fluorescence images at different time intervals are shown with the corresponding fluorescence intensity scale [5×10^7–2.0×10^8 (photons/s)/(μW/cm^2)].
